# Serum ferritin change rate combined with a multidimensional inflammation model for predicting efficacy and survival in extensive-stage small-cell lung cancer patients undergoing immunotherapy: a single-center, retrospective cohort study

**DOI:** 10.3389/fimmu.2026.1755851

**Published:** 2026-03-11

**Authors:** Yalan Liu, Xinfu Liu, Yurong Li, Mengjie Li, Yudong Su, Peng Chen

**Affiliations:** 1 Department of Thoracic Oncology, Tianjin Medical University Cancer Institute & Hospital, National Clinical Research Center for Cancer, Tianjin Key Laboratory of Cancer Prevention and Therapy, Tianjin’s Clinical Research Center for Cancer, Tianjin, China; 2 Department of Oncology, The Central Hospital of Shaoyang, Shaoyang, China

**Keywords:** efficacy, inflammation model, SCLC, serum ferritin, survival outcome

## Abstract

**Background:**

First-line immunotherapy combined with chemotherapy for extensive-stage small-cell lung cancer (ES-SCLC) has been consistently recommended by clinical guidelines, but the improvement in overall survival remains limited. There is an urgent need to identify reliable predictive biomarkers for immunotherapy to select patients who would benefit most. Serum ferritin (SF) is a key regulator in ferroptosis and plays a significant role in immunotherapy of lung cancer. Therefore, we hypothesized that the change rate of serum ferritin (ΔSF) during immunotherapy, combined with inflammation-related indicators, could serve as a useful predictive marker for treatment response in ES-SCLC patients.

**Methods:**

We comprehensively reviewed the medical records of 550 ES-SCLC patients, divided into an experimental group (425 patients receiving immune checkpoint inhibitors (ICIs) plus chemotherapy) and a control group (125 patients receiving chemotherapy alone). The study analyzed the correlation between pre-immunotherapy SF levels and molecular subtypes, clinical stage, tumor location, and programmed death-ligand 1 (PD-L1) expression in ES-SCLC patients; the correlation of SF levels and ΔSF with objective response rate (ORR); the correlation of ΔSF combined with a multidimensional inflammation model—including neutrophil-to-lymphocyte ratio (NLR), lactate dehydrogenase (LDH), and C-reactive protein (CRP)—with ORR; and survival analysis for these parameters.

**Results:**

Patients with lower SF levels before immunotherapy had a higher ORR (χ² = 4.837, *P* = 0.035) and longer progression-free survival (PFS) (median 6.9 vs. 4.1 months). Patients with a high ΔSF during immunotherapy showed a higher ORR (χ² = 6.475, *P* = 0.019). Patients with high ΔSF combined with low NLR and LDH levels before immunotherapy were more likely to achieve a higher ORR (*P* < 0.001). After integration, patients with low SF levels and high ΔSF before immunotherapy had the best PFS, whereas those with high SF levels and low ΔSF before immunotherapy had the worst PFS (median 8.9 vs. 4.5 months). Within the high ΔSF group, patients with lower NLR had longer PFS than those with higher NLR (median 9.8 vs. 5.2 months); similarly, patients with lower LDH levels had longer PFS than those with higher LDH levels (median 9.2 vs. 5.6 months). Multivariate analysis identified SF levels before immunotherapy (HR = 1.58, *P* = 0.026) and ΔSF during immunotherapy (HR = 0.52, *P* = 0.002) as independent prognostic factors. SCLC clinical stage (HR = 0.56, *P* = 0.037) and molecular subtype (SCLC-A: HR = 1.67, *P* = 0.003; SCLC-N: HR = 1.51, *P* = 0.012; SCLC-P: HR = 0.73, *P* = 0.004; SCLC-Y: HR = 0.64, *P* = 0.003) were also independent prognostic factors. However, NLR and LDH levels alone were not independent prognostic factors and required combined assessment with ΔSF.

**Conclusion:**

Our study suggests that the serum ferritin change rate combined with the NLR and LDH inflammation model can serve as a biomarker for predicting the efficacy and survival outcomes of immunotherapy in ES-SCLC.

## 1 Introduction

According to data from the International Agency for Research on Cancer (IARC), lung cancer has the highest global incidence and mortality rates among all cancers. In 2022, approximately 2.48 million new cases of lung cancer were diagnosed worldwide, with about 1.80 million deaths (1). The incidence of lung cancer has been steadily rising over the past decade, which is associated with factors such as industrialization, increased smoking rates, and environmental influences. Lung cancer is further classified into non-small cell lung cancer (NSCLC) and small cell lung cancer (SCLC). SCLC accounts for approximately 15% of all lung cancer cases, with a 5-year survival rate typically below 7%. It is an aggressive neuroendocrine carcinoma characterized by rapid tumor cell proliferation, high metastatic potential, a tendency to develop treatment resistance, and an extremely poor prognosis (2). More than two-thirds of SCLC patients are diagnosed at the extensive-stage, while about one-third are at the limited- stage. For a long time, etoposide combined with carboplatin has been the standard first-line treatment for ES-SCLC. In recent years, the incorporation of immunotherapy into treatment strategies, specifically chemotherapy combined with immunotherapy, has extended both the overall survival (OS) and progression-free survival (PFS) of ES-SCLC patients (3). Although immunotherapy has supplemented the relatively limited treatment options for ES-SCLC, clinical study data indicate that only about 15% of SCLC patients exhibit a response to immune checkpoint inhibitors (ICIs), and the overall benefit from immunotherapy for SCLC patients remains limited (4). Based on results from two large clinical trials, CASPIAN and IMpower133, the combination of chemotherapy and the immunotherapy agents atezolizumab or durvalumab—both programmed cell death ligand 1 (PD-L1) inhibitors, compared to control groups, demonstrated only a very modest extension in overall OS (approximately 2 months) (5, 6). Therefore, there is an urgent need to explore the characteristics of the SCLC patient subgroup that benefits from immunotherapy, to gain a deeper understanding of SCLC tumor heterogeneity, and to identify more biomarkers and independent prognostic factors to guide treatment.

Currently, clinically recommended biomarkers for predicting the efficacy of tumor immunotherapy include PD-L1 expression, microsatellite instability (MSI), and tumor mutational burden (TMB). PD-L1 is the most commonly used biomarker in immunotherapy of lung cancer. However, the level of PD-L1 expression alone is often insufficient for directly determining treatment efficacy (7). Even lung cancer patients with high PD-L1 expression may fail to respond to immunotherapy or may not achieve long-term clinical benefit, and cumulative immune-related adverse events can potentially be fatal. For SCLC, clinical guidelines do not routinely recommend PD-L1 testing for several key reasons. First, the immune microenvironment of SCLC is characterized by a low PD-L1 expression rate, high TMB, and an abundance of myeloid-derived suppressor cells (MDSCs) and regulatory T cells (Tregs), which facilitate PD-L1-independent immune escape mechanisms (8, 9). Second, clinical evidence indicates that the benefit from the combination of immunotherapy and chemotherapy is independent of PD-L1 status. Finally, from a practical standpoint, PD-L1 testing does not alter first-line treatment decisions and adds to healthcare costs. Consequently, there is a pressing need for more clinically useful biomarkers to predict the efficacy of immunotherapy in ES-SCLC.

Recent studies have discovered that ferroptosis is one of the important targets of immunotherapy (10). Serum ferritin (SF), a key metabolite in the ferroptosis process, serves as a marker of iron storage in the body, and its abnormal levels indicate iron metabolism dysregulation. Ferroptosis is a form of regulated cell death driven by iron-dependent accumulation of lipid peroxides. Its core defense system is glutathione peroxidase 4 (GPX4) (11), which utilizes glutathione to reduce toxic lipid peroxides into harmless lipid alcohols. Any factor that impairs GPX4 function or depletes glutathione (e.g., by inhibiting the cystine-glutamate antiporter system Xc⁻) can induce ferroptosis, and dysregulated iron metabolism is a key factor in triggering this process (12). Studies have found that in SCLC, inducing ferroptosis can not only directly kill tumor cells but also remodel the tumor immune microenvironment, thereby potentially enhancing the efficacy of immune checkpoint inhibitors (13). Elevated serum ferritin levels in SCLC are often associated with multiple factors. The tumor itself and the inflammatory state it triggers (e.g., high levels of IL-6 and C-reactive protein, elevated neutrophil-to-lymphocyte ratio, increased lactate dehydrogenase) stimulate the liver to synthesize and release ferritin, making it an acute-phase protein (14). During anti-tumor therapy, rapidly proliferating and apoptotic/necrotic tumor cells release their intracellular ferritin into the bloodstream, causing a transient increase in serum ferritin levels. To meet their own rapid proliferation demands, tumor cells upregulate transferrin receptors, among others, to enhance iron uptake, leading to alterations in the intracellular iron pool, which may also influence serum levels (15).

Since ferroptosis is an integral component of the immunotherapy pathway, we hypothesized that ferritin, much like PD-L1, could serve as an effective predictive indicator for selecting patients suitable for immunotherapy, while also accounting for confounding factors such as inflammation and iron overload due to blood product transfusions. We speculate that patients with low baseline ferritin levels and a low-inflammatory state should possess higher levels of free iron and exhibit a better response to immunotherapy. This study measured SF levels in ES-SCLC patients before and during treatment with immune checkpoint inhibitors, calculating the serum ferritin change rate (ΔSF). We investigated whether ΔSF, combined with a multidimensional inflammation model (NLR + LDH + CRP), can effectively predict the efficacy of tumor immunotherapy. Furthermore, this study explored the relationships between SF levels and PD-L1 expression, tumor location, clinical stage, molecular subtype, and their association with survival outcomes in ES-SCLC.

## 2 Methods

### 2.1 Patient selection

This study comprehensively collected data from 550 patients with pathologically confirmed ES-SCLC at Tianjin Medical University Cancer Institute & Hospital between January 1, 2020, and December 31, 2024. The patients were divided into an experimental group (425 patients receiving immunotherapy combined with chemotherapy) and a control group (125 patients receiving chemotherapy alone).

The collected data included sociodemographic characteristics such as sex, age, and smoking history.

Tumor-related information included TNM stage, tumor anatomical location, molecular subtype, treatment regimen, and number of treatment cycles. Hematological parameters included baseline serum ferritin level (before immunotherapy/chemotherapy), and SF levels measured after 4–6 treatment cycles (12–18 weeks). The ΔSF was calculated as follows: ΔSF = (Pre-treatment SF - On-treatment SF)/Pre-treatment SF × 100%. It also included neutrophil count, lymphocyte count, neutrophil-to-lymphocyte ratio (NLR), lactate dehydrogenase (LDH), and C-reactive protein (CRP) before treatment. Based on imaging reviews (e.g., contrast-enhanced CT of the chest/abdomen, contrast-enhanced MRI of the brain) conducted every two cycles, tumor response was assessed for each patient according to the Response Evaluation Criteria in Solid Tumors (RECIST) version 1.1, recorded as complete response (CR), partial response (PR), stable disease (SD), or progressive disease (PD). The ORR was calculated as (number of patients with CR + PR)/total number of patients × 100%. PFS was defined as the time from the initiation of immunotherapy to disease progression or the last follow-up. Patient follow-up data were obtained through medical record reviews and telephone surveys. All imaging evaluations were uniformly reported by the Imaging Center of Tianjin Medical University Cancer Institute & Hospital. All blood tests were performed in the Department of Clinical Laboratory at the same hospital. We recorded the PFS for each patient through medical record documentation and telephone follow-up. The follow-up period will continue until August 31, 2025. This study was conducted in compliance with the Declaration of Helsinki. This study is a retrospective, observational study based on electronic medical records. All data were analyzed after anonymization. The study protocol was reviewed and approved by the Ethics Review Committee of Tianjin Medical University Cancer Institute & Hospital (no.2025-03-224). Given the retrospective nature of this study, which involved no therapeutic interventions and solely utilized existing clinical, pathological, and imaging data, the Ethics Committee waived the requirement for obtaining individual patient informed consent.

### 2.2 Inclusion and exclusion criteria

Patients who met all of the following criteria were included in this study:

(1) Histologically or cytologically confirmed ES-SCLC. Extensive-stage was defined as disease extending beyond one hemithorax or accompanied by distant metastases (according to the Veterans Administration Lung Study Group staging system or TNM stage IV).

(2) Had a defined treatment regimen.

* Experimental Group: Patients received at least 4 cycles of first-line immunotherapy combined with chemotherapy. Immunotherapeutic agents were immune checkpoint inhibitors, such as anti-PD-1/PD-L1 antibodies (including NMPA-approved drugs like durvalumab, atezolizumab, serplulimab, adebrelimab, tislelizumab, toripalimab, etc.). Chemotherapy consisted of platinum-based doublet regimens (e.g., etoposide plus cisplatin or carboplatin).

* Control Group: Patients received at least 4 cycles of first-line platinum-based doublet chemotherapy and did not receive combined immunotherapy.

(3) Possessed complete baseline sociodemographic characteristics and tumor clinicopathological features. Had baseline SF levels before immunotherapy or chemotherapy, as well as serum ferritin levels during treatment cycles 4~6 (weeks 12~18), to allow calculation of ΔSF. Had baseline pre-treatment hematological parameters, such as neutrophil and lymphocyte counts, for calculating the neutrophil-to-lymphocyte ratio. Had regular imaging evaluations according to RECIST 1.1 criteria, usable for efficacy assessment and PFS calculation.

(4) Had follow-up data obtained through medical record systems or telephone follow-up containing at least progression-free survival information.

Patients meeting any of the following criteria were excluded:

(1) Those with severely missing or incomplete clinical medical records, failing to meet the aforementioned “data completeness” requirements.

(2) Those with a concurrent active malignancy that could interfere with efficacy and survival analysis.

(3) Those who discontinued immunotherapy or chemotherapy prematurely for any reason or changed their treatment regimen before the planned cycle completion, preventing standardized efficacy evaluation and dynamic monitoring of SF.

(4) Those who received thoracic radiotherapy or any other local ablative therapy during the first-line treatment period (prior to disease progression) were excluded.

(5) Those missing key baseline or on-treatment serum ferritin measurements, baseline hematological parameters, or regular imaging follow-up data, making it impossible to calculate ΔSF or PFS.

(6) Those with comorbid conditions that could significantly affect SF levels, such as acute or chronic liver disease, hematological disorders, active infections, severe rheumatic autoimmune diseases, etc., or those who had received blood product transfusions within the past 3 months.

(7) Those lost to follow-up before the data cutoff date, making it impossible to ascertain their survival status and PFS information.

### 2.3 Detection methods

SF levels were measured using a human ferritin kit (Zhongxiu Science and Technology, CO.LTD, Xinxiang, China) via a chemiluminescent particle immunoassay. The expression level of PD-L1 in corresponding paraffin-embedded tumor tissues was detected using immunohistochemistry (IHC) and assessed by experienced pathologists (PD-L1 IHC 22C3 pharmDx, Beijing, China). The molecular subtypes of SCLC were determined by detecting the expression of four proteins—ASCL1, NEUROD1, POU2F3, and YAP1—using IHC with specific antibodies on tumor tissue sections. Neutrophil count, lymphocyte count, NLR and CRP were measured using the colloidal gold immunochromatography/colorimetric method. LDH was quantitatively detected via a coupled enzyme reaction.

### 2.4 Statistical analysis

Statistical analysis was performed using SPSS software (version 27.0) and R software (version 4.2.3; R Project for Statistical Computing). The median value of SF levels was defined as the cutoff point. Baseline SF was defined as the SF value measured at the same center within 14 days before treatment initiation. The serum ferritin change rate (ΔSF) was calculated as follows: ΔSF = (Pre-treatment SF - On-treatment SF)/Pre-treatment SF × 100%. A ΔSF value ≥ 15% was considered indicative of SF downregulation, defining the high ΔSF group; a ΔSF value ≤ -15% was considered indicative of SF upregulation, defining the low ΔSF group; and a ΔSF value between -15% and +15% was judged as the no significant change group.

Box plots and the non-parametric Mann-Whitney U test were used to analyze the distribution of SF levels across patients with different PD-L1 expression statuses, molecular subtypes, and clinical stages. Survival curves were plotted using the Kaplan-Meier method and compared with the log-rank test. The correlation between SF levels (and ΔSF) and the ORR was assessed using Pearson’s correlation coefficient. Univariate and multivariate analyses for PFS were conducted using the Cox proportional hazards model to identify independent prognostic factors. A *p*-value < 0.05 was considered statistically significant.

## 3 Results

### 3.1 Baseline characteristics of the patients

This study included a total of 550 patients, divided into an experimental group (n=425) and a control group (n=125). The experimental group comprised 302 males and 123 females, while the control group included 87 males and 38 females. In the experimental group, 265 patients were aged ≤65 years and 160 were >65 years, with a median age of 62 years (range: 41~78). In the control group, 78 patients were aged ≤65 years and 47 were >65 years, with a median age of 58 years (range: 38~76). Regarding smoking history, the experimental group included 162 non-smokers, 178 patients with a smoking history of ≤30 pack-years, and 85 patients with >30 pack-years. The control group included 76 non-smokers, 24 patients with ≤30 pack-years, and 25 patients with >30 pack-years. Pack-years were calculated as the number of packs smoked per day multiplied by the number of years smoked.

Based on the anatomical location of the primary tumor, the experimental group included 228 central, 157 peripheral, and 43 diffuse types, while the control group included 75 central, 34 peripheral, and 16 diffuse types. According to the TNM staging for ES-SCLC, M1a denotes intrathoracic metastasis, M1b single extrathoracic organ metastasis, and M1c multiple extrathoracic organ metastases. In the experimental group, 257 cases were classified as M1a, 109 as M1b, and 59 as M1c. In the control group, 85 cases were M1a, 13 were M1b, and 27 were M1c.In 2019, Gay et al. (16) proposed a molecular subtyping classification for SCLC based on the relative expression levels of four key transcription factors: ASCL1, NEUROD1, YAP1, and POU2F3. Accordingly, SCLC is categorized into four subtypes: SCLC-A, SCLC-N, SCLC-P, and SCLC-Y, defined by the highest expression of the respective transcription factor. In the experimental group, the distribution of these subtypes was 218, 95, 72, and 40 cases, respectively. In the control group, the distribution was 77, 21, 15, and 12 cases, respectively. The baseline characteristics of the enrolled patients are presented in **Table 1**. The types and numbers of immune checkpoint inhibitors used in this study are summarized in **Table 2**.

**Table 1:** Demographic and clinicopathological characteristics of patients.

Patient characteristics	Experimental group No. (%) (N = 425)	Control group No. (%) (N = 125)
Sex		
Male	302(71.06)	87(69.60)
Female	123(28.94)	38(30.40)
Age (years)		
≤65	265(62.35)	78(62.40)
>65	160(37.65)	47(37.60)
Mean age	62 years old	58 years old
Smoking Status		
Non-smoker	162(38.12)	76(60.80)
≤ 30 pack-years	178(41.88)	24(19.20)
>30 pack-years	85(20.00)	25(20.00)
Tumor Location		
Central type	228(53.65)	75(60.00%)
Peripheral type	157(36.94)	34(27.20%)
Diffuse type	43(10.11)	16(12.80%)
Clinical Stage		
M1a (Stage IVa)	257(60.47)	85(68.00)
M1b(Stage IVa)	109(25.65)	13(10.40)
M1c(Stage IVb)	59(13.88)	27(21.60)
Molecular Subtype		
SCLC-A	218(51.29)	77(61.60)
SCLC-N	95(22.35)	21(16.80)
SCLC-P	72(16.95)	15(12.00)
SCLC-Y	40(9.41)	12(9.60)

**Table 2:** The number and proportion of PD-1/PD-L1 inhibitors used in this study.

PD-1/PD-L1 inhibitors	No. (%) (N = 425)
PD-L1 inhibitors	
Durvalumab	56(13.18)
Atezolizumab	45(10.59)
PD-1 inhibitors	
Tislelizumab	165(38.83)
Serplulimab	78(18.35)
Toripalimab	53(12.47)
Adebrelimab	28(6.58)

PD-1, programmed cell death-1; PD-L1, programmed cell death-ligand 1.

### 3.2 Serum ferritin levels

In the experimental group, the pre-treatment SF levels before chemotherapy and immunotherapy ranged from a minimum of 54.31 μg/L to a maximum of 6743.43 μg/L. The 25th percentile, median, and 75th percentile values were 126.43 μg/L, 259.85 μg/L, and 469.86 μg/L, respectively. In the control group, the pre-chemotherapy serum ferritin levels ranged from 43.75 μg/L to 5426.61 μg/L, with the 25th percentile, median, and 75th percentile values being 132.49 μg/L, 267.84 μg/L, and 413.26 μg/L, respectively.

The ΔSF was calculated as follows: ΔSF = (Pre-treatment SF - On-treatment SF)/Pre-treatment SF × 100%. A ΔSF value ≥ 15% was defined as SF downregulation (high ΔSF group); a ΔSF value ≤ -15% was defined as SF upregulation (low ΔSF group); and a ΔSF value between -15% and +15% was defined as the no significant change group. In the experimental group, 217 patients were classified into the high ΔSF group, 163 patients into the low ΔSF group, and 45 patients into the no significant change group. In the control group, the corresponding numbers were 56, 54, and 15 patients, respectively.

### 3.3 Analysis of the correlation between pre-treatment SF levels and SCLC molecular subtypes, clinical stage, tumor location, and PD-L1 expression

**Figure 1:**
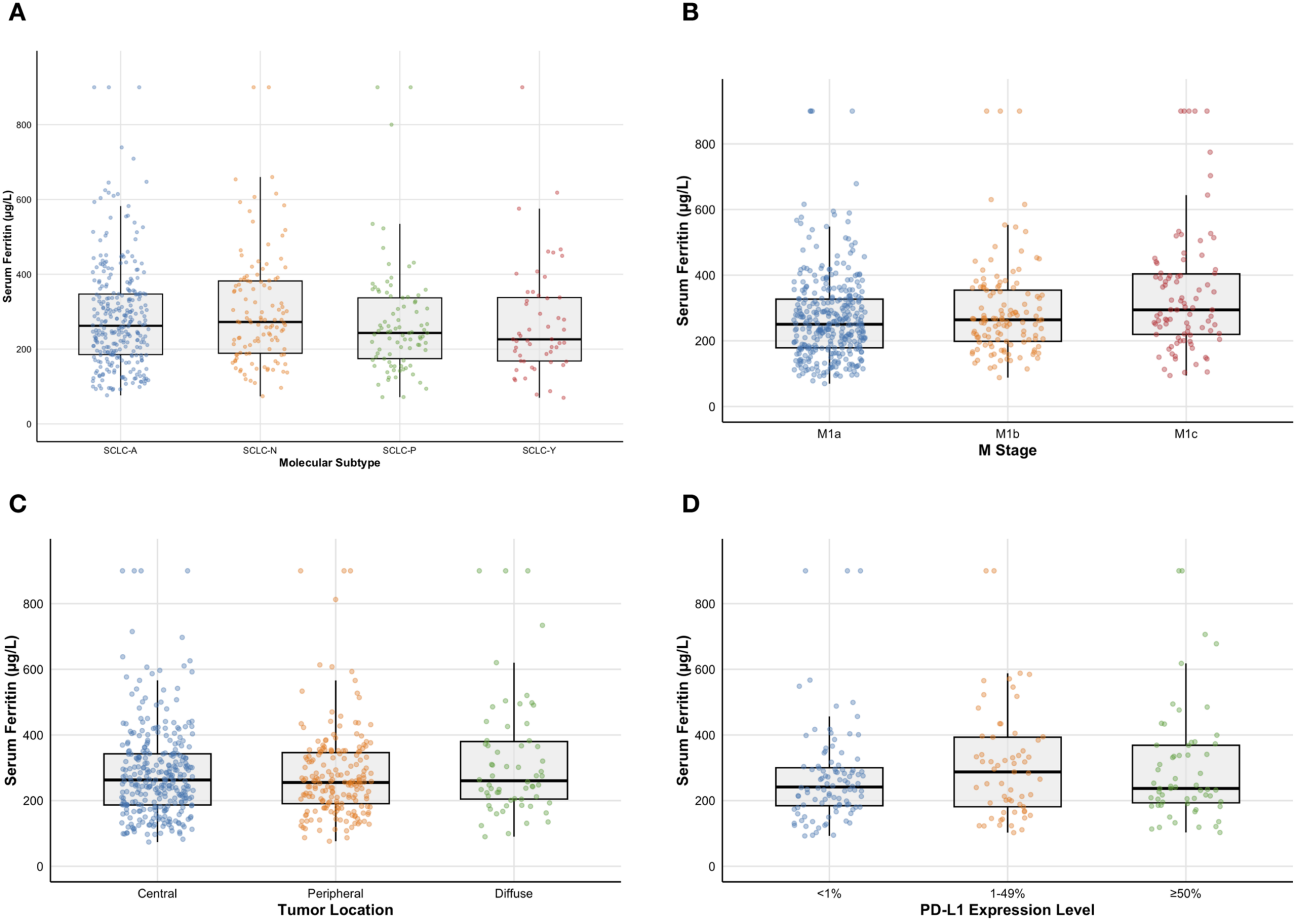
**(a)** Distribution of serum ferritin levels across SCLC molecular subtypes (SCLC-A, n=295; SCLC-N, n=116; SCLC-P, n=87; SCLC-Y, n=52). The results indicate no significant correlation between molecular subtype and serum ferritin level (non-parametric rank-sum test, *P* = 0.615). **(b)** Distribution of serum ferritin levels across SCLC M stages (M1a, n=342; M1b, n=122; M1c, n=86). The results show no statistically significant correlation between tumor stage and serum ferritin level, although the P-value approached 0.05 (non-parametric rank-sum test, P = 0.058). **(c)** Distribution of serum ferritin levels based on the anatomical location of the primary SCLC tumor (central, peripheral, diffuse). The results reveal no correlation between tumor location and serum ferritin level (non-parametric rank-sum test, P = 0.314). **(d)** Distribution of serum ferritin levels across different PD-L1 expression ranges (PD-L1 < 1%, n=97; PD-L1 ≥1% and <50%, n=59; PD-L1 ≥50%, n=57). No correlation was found between PD-L1 expression and serum ferritin level (non-parametric rank-sum test, P = 0.541).

Patients (n=550) were categorized into four groups based on their molecular subtypes (SCLC-A, SCLC-N, SCLC-P, SCLC-Y), and the distribution of their pre-treatment SF levels across these groups is presented using box plots (**Figure 1a**). Fifteen cases with SF levels > 900 μg/L were defined as 900 μg/L in the box plots. The results indicated no significant correlation between molecular subtype and SF levels (non-parametric rank-sum test, *P* = 0.615).

Patients were divided into three groups according to their M stage (M1a, M1b, M1c). The distribution of SF levels across these three groups is displayed in box plots (**Figure 1b**). Fifteen cases with SF levels > 900 μg/L were defined as 900 μg/L in the box plots. The results showed no statistically significant correlation between tumor M stage and SF levels, although the *P*-value approached 0.05 (non-parametric rank-sum test, *P* = 0.058). The box plots suggest that the baseline SF levels in SCLC patients with M1a and M1b disease were distributed lower than those in patients with M1c disease. It is generally recognized that SCLC patients with stage IVb disease (distant extranthoracic metastases) have poorer treatment responses and survival compared to those with stage IVa disease. Therefore, lower baseline SF levels in SCLC patients may be associated with better treatment response and longer survival, which is consistent with our initial hypothesis.

Patients were categorized into three groups based on the anatomical location of the primary tumor (central, peripheral, diffuse). The distribution of SF levels across these three groups is shown in box plots (**Figure 1c**). Fifteen cases with SF levels > 900 μg/L were defined as 900 μg/L in the box plots. The results revealed no correlation between tumor location and SF levels (non-parametric rank-sum test, *P* = 0.314).

We collected 213 cases with available PD-L1 expression results. Patients were divided into three groups based on PD-L1 expression levels (PD-L1 < 1%; PD-L1 ≥1% and <50%; PD-L1 ≥50%). The association between SF and PD-L1 expression is illustrated using a box plot (**Figure 1d**). No correlation was found between PD-L1 expression and SF levels (non-parametric rank-sum test, *P* = 0.541).

### 3.4 Correlation between SF levels, ΔSF, and ORR

**Figure 2:**
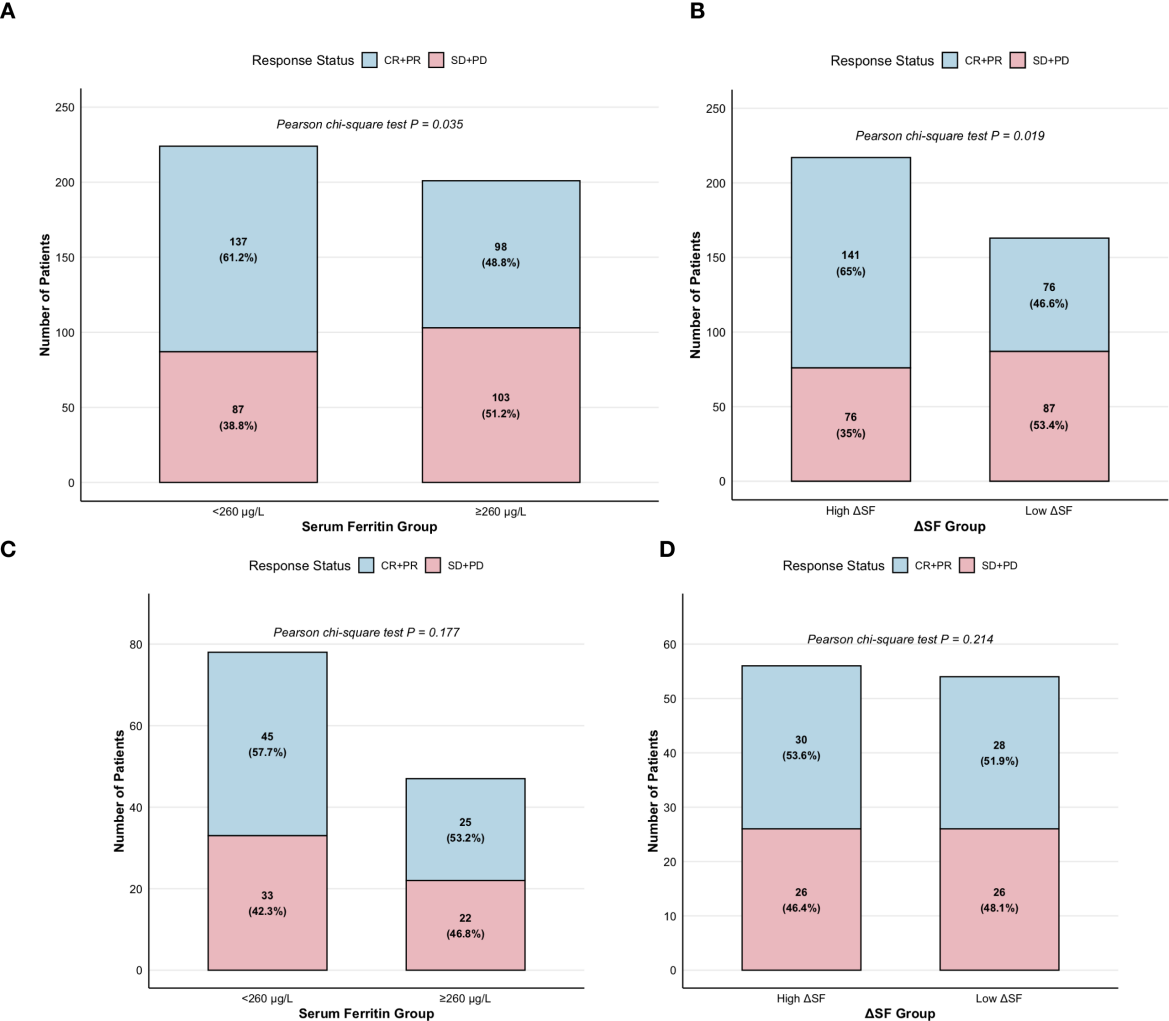
**(a)** Histogram of ORR in 425 patients from the experimental group, stratified by pre-treatment serum ferritin levels. The ORR was 61.2% (137/224) in the group with serum ferritin < 260 μg/L and 48.7% (98/201) in the group with serum ferritin > 260 μg/L. The group with lower pre-treatment serum ferritin levels demonstrated a superior ORR compared to the group with higher levels (Pearson’s chi-square test, χ² = 4.837, *P* = 0.035). **(b)** Histogram of ORR in 380 patients from the experimental group, categorized by the defined ΔSF during treatment. The ORR was 64.9% (141/217) in the high ΔSF group and 46.6% (76/163) in the low ΔSF group. The high ΔSF group showed a significantly better ORR than the low ΔSF group during chemoimmunotherapy (χ² = 6.475, P = 0.019). **(c)** Histogram of ORR in all 125 patients from the control group, stratified by pre-treatment serum ferritin levels. The ORR was 57.7% (45/78) in the group with serum ferritin < 260 μg/L and 53.2% (25/47) in the group with serum ferritin > 260 μg/L. No significant correlation was observed between pre-treatment SF levels and ORR (Pearson’s chi-square test, χ² = 4.837, P = 0.177). **(d)** Among 110 patients in the control group, the ORR was 53.6% (30/56) in the high ΔSF group and 51.9% (28/54) in the low ΔSF group. No significant association was found between the level of ΔSF and ORR during chemotherapy alone (Pearson’s chi-square test, χ² = 5.421, P = 0.214).

The 425 patients in the experimental group were divided into two groups using a pre-treatment SF cutoff of 260 μg/L. The correlation between serum ferritin and ORR was analyzed using Pearson’s correlation coefficient (Pearson chi-square test, χ² = 4.837, *P* = 0.035) (**Figure 2a**). The ORR was 61.2% (137/224) in the group with SF < 260 μg/L and 48.7% (98/201) in the group with SF > 260 μg/L. This suggests that the group with lower pre-treatment SF levels had a superior ORR compared to the group with higher levels. Based on the defined ΔSF during treatment, the 380 eligible patients were categorized into a high ΔSF group (n=217) and a low ΔSF group (n=163). A similar analysis was performed (Pearson chi-square test, χ² = 6.475, *P* = 0.019) (**Figure 2b**). The ORR was 64.9% (141/217) in the high ΔSF group and 46.6% (76/163) in the low ΔSF group. This indicates that the high ΔSF group had a significantly better ORR than the low ΔSF group during chemotherapy and immunotherapy. To exclude the potential influence of chemotherapy alone on ΔSF and ORR, we further analyzed the correlation between pre-chemotherapy SF levels, ΔSF, and ORR in the control group. We have a comprehensive supplementary table (**Supplementary Table 1**) that presents, for each clinical and molecular subgroup, the absolute counts and percentages of patients classified as responders (CR + PR) and non-responders (SD + PD).

The 125 patients in the control group were also divided into two groups using the same pre-treatment serum ferritin cutoff of 260 μg/L. The ORR was 57.7% (45/78) in the group with serum ferritin < 260 μg/L and 53.2% (25/47) in the group with serum ferritin > 260 μg/L. Analysis using Pearson’s correlation coefficient showed no significant correlation between pre-treatment SF levels and ORR (Pearson chi-square test, χ² = 4.837, *P* = 0.177) (**Figure 2c**). Among the 110 eligible patients in the control group, the ORR was 53.6% (30/56) in the high ΔSF group and 51.9% (28/54) in the low ΔSF group. Analysis of the correlation between ΔSF and ORR yielded similar results (Pearson chi-square test, χ² = 5.421, *P* = 0.214), indicating no significant association between ΔSF level and ORR during chemotherapy alone (**Figure 2d**).

These results demonstrate that, after excluding the interference of the chemotherapy factor, lower pre-immunotherapy SF levels and a higher ΔSF during immunotherapy are associated with a better disease response, which is consistent with our initial hypothesis. To further validate our hypothesis, we investigated the correlation between serum ferritin levels and patient prognosis.

### 3.5 Correlation between the multidimensional inflammation model combined with ΔSF and ORR

Our analysis indicated that the high ΔSF group had a superior ORR compared to the low ΔSF group during chemotherapy and immunotherapy in the experimental group. However, could the treatment efficacy in these patients be influenced by systemic inflammatory factors? Therefore, we collected data on ΔSF levels, pre-treatment NLR, LDH, and CRP from these 380 patients. The correlation between the inflammation model combined with ΔSF and ORR was assessed using the non-parametric rank-sum test.

Patients were divided into two groups based on their ΔSF levels. In the high ΔSF group, the median NLR of patients who achieved response (141/217) was 2.9 (interquartile range [IQR]: 2.1~4.2), which was significantly lower than that of the non-responders (76/217), who had a median NLR of 5.3 (IQR: 4.1~7.5; *P* < 0.001). In the low ΔSF group, the median NLR of responders (76/163) was 4.3 (IQR: 3.4~5.1), showing no statistically significant difference compared to the median NLR of 4.5 (IQR: 3.9~5.5) in non-responders (87/163; *P* = 0.214). Similarly, in the high ΔSF group, the median LDH of responders (141/217) was 145 U/L (IQR: 126.6~156.4 U/L), significantly lower than that of non-responders (76/217), who had a median LDH of 278 U/L (IQR: 245.7~302.4 U/L; *P* < 0.001). In the low ΔSF group, the median LDH of responders (76/163) was 213 U/L (IQR: 198.7~253.6 U/L), which was not significantly different from the median LDH of 225.6 U/L (IQR: 201.3~248.7 U/L) in non-responders (87/163; *P* = 0.874). When analyzing CRP using the same method, we found that CRP did not significantly affect the ORR regardless of the ΔSF level (*P* > 0.05). Therefore, we conclude that patients with high ΔSF combined with low pre-treatment NLR and LDH are more likely to achieve an objective response.

### 3.6 Survival analysis

**Figure 3:**
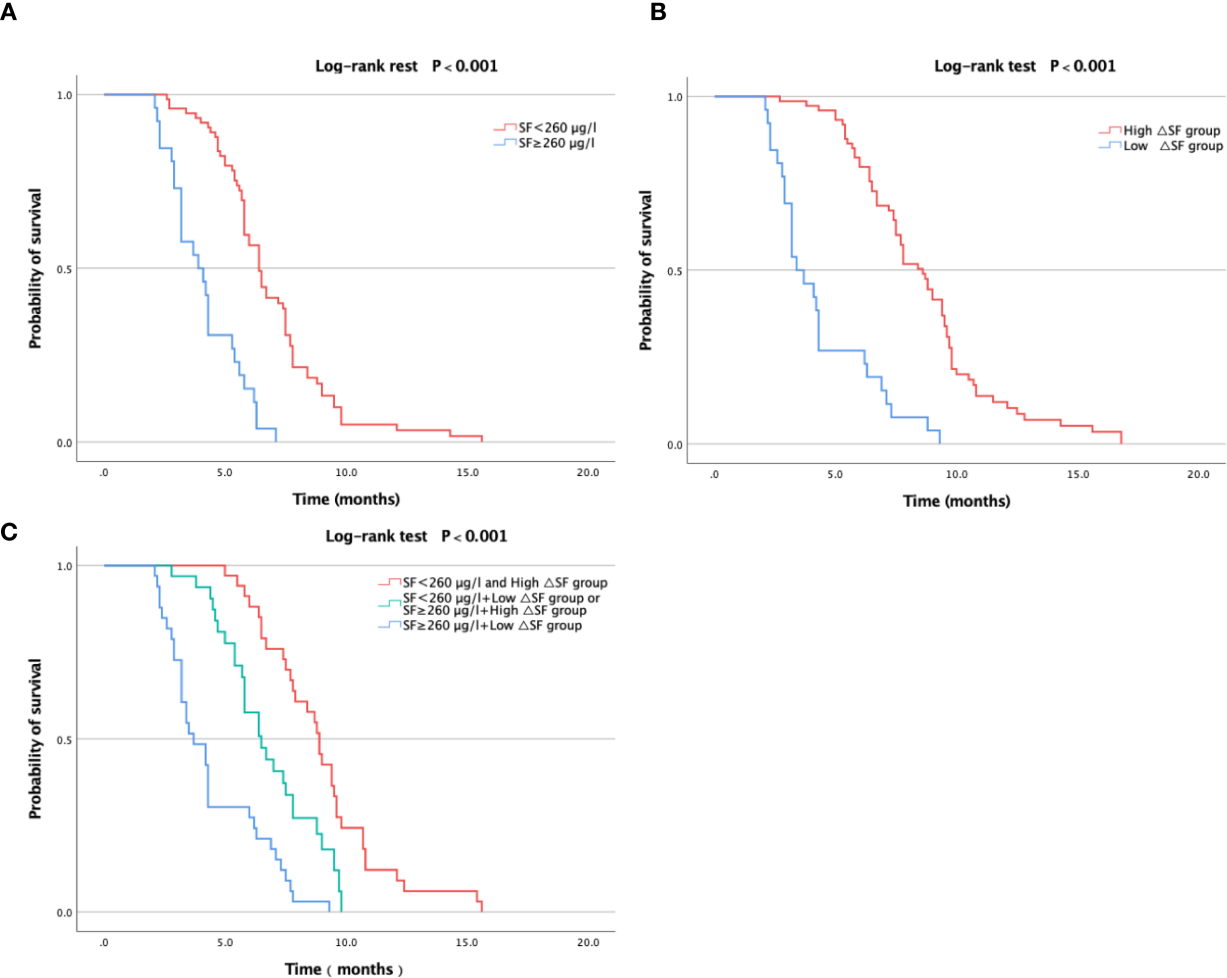
Survival curves for PFS. **(a)** The 425 patients in the experimental group were divided into two groups using a pre-immunotherapy SF cutoff of 260 μg/L. The results showed that patients with low pre-immunotherapy SF levels had a longer PFS than those with high levels (median PFS: 6.9 months vs. 4.1 months; log-rank test, *P* < 0.001). **(b)** The 380 eligible patients were categorized into high and low ΔSF groups. The results demonstrated that during immunotherapy, patients in the high ΔSF group had a longer PFS than those in the low ΔSF group (median: 8.6 months vs. 4.3 months; log-rank test, P < 0.001) **(c)** Patients with low pre-immunotherapy SF levels and high ΔSF had the best PFS, whereas those with high pre-immunotherapy SF levels and low ΔSF had the worst PFS (median: 8.9 months vs. 4.5 months; log-rank test, P < 0.001).

The median PFS for all patients was 6.1 months. The 425 patients in the experimental group were divided into two groups using a pre-immunotherapy SF cutoff of 260 μg/L. The results showed that patients with low pre-immunotherapy SF levels had a longer PFS than those with high levels (median PFS: 6.9 months vs. 4.1 months; log-rank test, *P* < 0.001). The corresponding survival curves are shown in **Figure 3a**.

. According to the defined criteria, the 380 eligible patients were categorized into high and low ΔSF groups. The results demonstrated that during immunotherapy, patients in the high ΔSF group had a longer PFS than those in the low ΔSF group (median: 8.6 months vs. 4.3 months; log-rank test, *P* < 0.001). The corresponding survival curves are shown in **Figure 3b**. Subsequently, we performed survival analysis using a combination of these two prognostic factors. Patients with low pre-immunotherapy SF levels and high ΔSF had the best PFS, whereas those with high pre-immunotherapy SF levels and low ΔSF had the worst PFS (median: 8.9 months vs. 4.5 months; log-rank test, *P* < 0.001). The corresponding survival curves are shown in **Figure 3c**.

**Figure 4:**
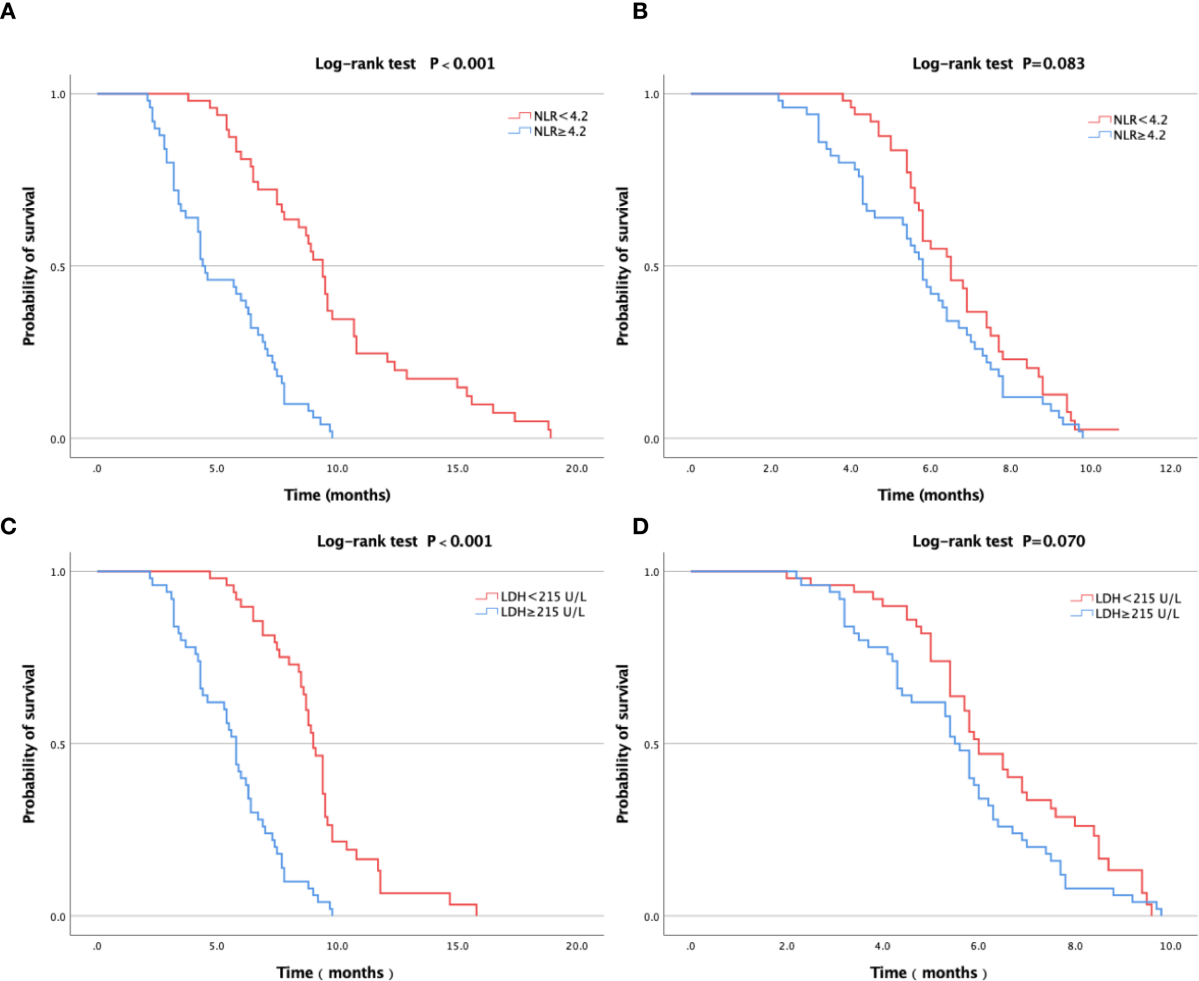
Survival curves for PFS. **(a)** Using a cutoff of NLR = 4.2, within the high ΔSF group, patients with lower NLR had a significantly longer PFS than those with higher NLR (median: 9.8 months vs. 5.2 months; log-rank test, P < 0.001). **(b)** Within the low ΔSF group, there was no statistically significant difference in PFS between patients with high and low NLR (median: 6.7 months vs. 5.8 months; log-rank test, P = 0.083). **(c)** Using a cutoff of LDH = 215 U/L, within the high ΔSF group, patients with lower LDH had a significantly longer PFS than those with higher LDH (median: 9.2 months vs. 5.6 months; log-rank test, P < 0.001). **(d)** Within the low ΔSF group, the difference in PFS between patients with high and low LDH was not statistically significant (median: 6.4 months vs. 5.5 months; log-rank test, P = 0.070).

Furthermore, we conducted survival analysis on the relationship between ΔSF during immunotherapy and the inflammation model (NLR and LDH) in the 380 SCLC patients. Using a cutoff of NLR = 4.2, within the high ΔSF group, patients with lower NLR had a significantly longer PFS than those with higher NLR (median: 9.8 months vs. 5.2 months; log-rank test, *P* < 0.001). The survival curves are shown in **Figure 4a**. In contrast, within the low ΔSF group, there was no statistically significant difference in PFS between patients with high and low NLR (median: 6.7 months vs. 5.8 months; log-rank test, *P* = 0.083). The survival curves are shown in **Figure 4b**. Similarly, using a cutoff of LDH = 215 U/L, within the high ΔSF group, patients with lower LDH had a significantly longer PFS than those with higher LDH (median: 9.2 months vs. 5.6 months; log-rank test, *P* < 0.001). The survival curves are shown in **Figure 4c**. Within the low ΔSF group, the difference in PFS between patients with high and low LDH was not statistically significant (median: 6.4 months vs. 5.5 months; log-rank test, *P* = 0.070). The survival curves are shown in **Figure 4d**.

Multivariate analysis of the 425 patients in the experimental group (Model 1, including baseline SF levels only) revealed that the pre-immunotherapy SF level was an independent factor affecting patient prognosis (HR = 1.58, *P* = 0.026). The clinical stage of SCLC (HR = 0.56, *P* = 0.037) and molecular subtype (SCLC-A: HR = 1.45, *P* = 0.004; SCLC-N: HR = 1.32, *P* = 0.017; SCLC-P: HR = 0.42, *P* = 0.023; SCLC-Y: HR = 0.56, *P* = 0.008) were also independent prognostic factors. Patient age, sex, smoking history, and tumor location were not associated with prognosis. Detailed information is included in **Table 3**.

**Table 3:** Details of the Cox proportional hazard model for 425 patients.

Variable	Univariate analysis			Multivariate analysis		
	HR	95%CI	*P*	HR	95%CI	*P*
Sex	0.76	0.45 ~1.32	0.314			
Age(years)	0.64	0.41~1.27	0.286			
Smoking status	1.34	0.89~2.45	0.276			
Tumor location	0.61	0.36~1.23	0.054			
Serum ferritin levels before treatment	1.65	1.05~2.31	0.024	1.58	1.12~2.09	0.026
Stage IVA/IVB	0.45	0.32~0.87	0.036	0.56	0.23~0.83	0.037
Molecular subtype						
SCLC-A	1.36	1.17~1.73	0.005	1.45	1.16~1.62	0.004
SCLC-N	1.48	1.21~1.89	0.008	1.32	1.01~1.73	0.017
SCLC-P	0.64	0.33~1.23	0.007	0.42	0.23~0.87	0.023
SCLC-Y	0.78	0.43~1.46	0.012	0.56	0.33~0.96	0.008

HR, hazard ratio; 95% CI = 95% confidence interval; SCLC, small-cell lung cancer.

Similarly, multivariate analysis of the 380 patients from the experimental group with available ΔSF data (Model 2, including ΔSF, NLR, and LDH) showed that ΔSF (HR = 0.52, *P* = 0.002), the clinical stage of SCLC (HR = 0.63, *P* = 0.019), and molecular subtype (SCLC-A: HR = 1.67, *P* = 0.003; SCLC-N: HR = 1.51, *P* = 0.012; SCLC-P: HR = 0.73, *P* = 0.004; SCLC-Y: HR = 0.64, *P* = 0.003) were independent factors affecting patient prognosis. However, NLR and LDH levels alone were not independent prognostic factors and needed to be evaluated in combination with the ΔSF level. Detailed information is contained in **Table 4**.

**Table 4:** Details of the Cox proportional hazard model for 380 patients.

Variable	Univariate analysis			Multivariate analysis		
	HR	95%CI	*P*	HR	95%CI	*P*
△SF	0.34	0.17~0.63	<0.001	0.52	0.29~0.79	0.002
NLR	2.32	1.71~2.89	0.004	3.16	2.71~3.89	0.012
LDH	2.57	2.13~2.83	0.015	2.68	2.24~3.01	0.031
Stage IVA/IVB	0.76	0.43~1.46	0.036	0.63	0.37~1.07	0.019
Molecular subtype						
SCLC-A	1.36	1.17~1.73	0.005	1.67	1.21~1.85	0.003
SCLC-N	1.48	1.21~1.89	0.008	1.51	1.15~1.67	0.012
SCLC-P	0.64	0.33~1.23	0.007	0.73	0.32~0.96	0.004
SCLC-Y	0.78	0.43~1.46	0.012	0.64	0.23~0.84	0.003

HR, hazard ratio; 95% CI = 95% confidence interval; SCLC, small-cell lung cancer; SF, serum ferritin; NLR, neutrophil-to-lymphocyte ratio; LDH, lactate dehydrogenase.

## 3 Discussion

Our study demonstrates that elevated SF levels in patients are often associated with disease progression and decreased survival in ES-SCLC. ES-SCLC patients with higher pre-immunotherapy SF levels had a poorer prognosis, whereas patients whose SF levels decreased during immunotherapy showed better treatment efficacy. Ferritin is a protein with multifunctional properties, playing significant roles in promoting cell proliferation, angiogenesis, and immune suppression (17). Numerous studies have detected high levels of ferritin in the serum of cancer patients, indicating a strong correlation with the extent of disease progression and prognosis (18). Therefore, we propose that ferritin also acts as a tumor marker. SF can exhibit non-specific elevation in certain conditions, including chronic diseases, inflammation, and malignant tumors (19). Reported evidence indicates elevated SF levels in various malignancies such as renal cancer, colorectal cancer, lung cancer, prostate cancer, and liver cancer. For instance, SF expression levels are significantly lower in healthy individuals compared to patients with advanced NSCLC. Colorectal cancer patients with higher SF levels after palliative surgery have shorter survival times (20). For hepatocellular carcinoma patients undergoing radiofrequency ablation, especially in cases that are alpha-fetoprotein-negative, SF can serve as a reference indicator for assessing survival time and recurrence (21).

Based on the scientific evidence presented above, we demonstrate for the first time that the baseline SF level before immunotherapy and its change rate (ΔSF) during treatment are independent prognostic factors in patients with ES-SCLC. The combination of ΔSF with a multidimensional inflammation model (NLR and LDH) can be used to predict the response to immunotherapy. Patients with lower pre-immunotherapy SF levels and those with a higher ΔSF (≥15%) during immunotherapy exhibited longer PFS and a higher ORR. Patients characterized by a higher ΔSF combined with lower pre-treatment NLR and LDH were more likely to achieve an objective response. We have also shown that considering these prognostic factors may assist in estimating survival time or time to recurrence. These findings collectively suggest that ΔSF combined with NLR and LDH holds significant clinical value for predicting the efficacy of immunotherapy in patients with ES-SCLC.

A key finding of our study is that NLR and LDH were only significant predictors of outcome when combined with ΔSF. This observation points to a fundamental biological synergy between systemic inflammation and iron metabolism in shaping the response to immunotherapy. We propose that NLR and LDH capture the ‘inflammatory context’—the presence and metabolic activity of immune cells and tumor burden—while ΔSF reflects the ‘execution phase’—the active engagement of iron-dependent ferroptosis as a terminal effector mechanism of anti-tumor immunity. In patients with high ΔSF, the inflammatory state (NLR/LDH) determines whether this iron utilization translates into effective tumor control: low NLR/LDH indicates a coordinated, productive immune response, whereas high NLR/LDH suggests that inflammation is dysregulated and fails to couple with ferroptosis. In contrast, in patients with low ΔSF, the inflammatory state is irrelevant because the ferroptosis effector pathway is not engaged. This conceptual framework explains the statistical interaction observed in our data and highlights the importance of multi-dimensional biomarkers that capture both the immune context and the terminal effector mechanisms of immunotherapy. Future studies should investigate whether iron-modulating therapies could convert low-ΔSF patients into high-ΔSF responders, thereby unlocking the predictive value of inflammatory markers in a broader population.

In 2020, Acheampong et al. (22) conducted the first comprehensive meta-analysis of PD-L1 expression in SCLC. Their study, after excluding studies with significant outliers, showed a PD-L1 positivity rate of 22.0%. Overall, PD-L1 expression levels are lower in SCLC compared to non-small cell lung cancer (NSCLC). PD-L1 expression levels were significantly correlated with IHC cutoff values and staining patterns, as well as geographical regions, but showed no significant correlation with different diagnostic antibodies or sample sizes. This study also proposed circulating tumor cells (CTCs) as an alternative “liquid biopsy specimen” to primary tumors. Recent studies (23) have indicated that PD-L1 expression on CTCs can assess the efficacy of PD-1/PD-L1 monoclonal antibody therapy in NSCLC patients. Evaluating PD-L1 expression in CTCs for SCLC might overcome the heterogeneity of PD-L1 expression (24, 25). Another biomarker with potential predictive value for lung cancer immunotherapy is tumor mutational burden (TMB). However, in large phase III trials of first-line immunotherapy combined with chemotherapy for SCLC (e.g., Keynote-604) (26), TMB failed to significantly predict treatment efficacy, suggesting that TMB might be more applicable for predicting efficacy in second-line and later-line immunotherapy settings (27). Multiple studies have shown that SCLC patients with high TMB, when treated with immunotherapy monotherapy (e.g., nivolumab) or combination immunotherapy (e.g., nivolumab + ipilimumab), have significantly better ORR, PFS, and OS compared to patients with low TMB (28, 29). For instance, the CheckMate 032 study (30) reported an ORR of 46.2% for combination therapy in high-TMB patients, compared to only 22.2% in low-TMB patients. Nevertheless, TMB as a predictive biomarker for immunotherapy has its limitations, primarily for the following reasons. First, SCLC exhibits high intra-tumoral and inter-tumoral heterogeneity. TMB test results can be influenced by the tumor sampling site, sample quality, and other factors, potentially leading to inaccurate reflection of the overall tumor immunogenicity in some patients. Second, the TMB biomarker alone is insufficient for comprehensively predicting immunotherapy efficacy. It needs to be combined with multi-dimensional information such as PD-L1 expression and immune microenvironment features (e.g., T-cell infiltration, inflammatory gene expression profiles) to improve predictive accuracy (31). Future research needs to further optimize TMB detection techniques and explore multi-biomarker combined models to enable more precise immunotherapy decision-making.

To date, the molecular mechanisms underlying SCLC pathogenesis have not been fully elucidated. SCLC involves different molecular pathways compared to NSCLC. Genomic analyses have revealed key genetic alterations in SCLC, with approximately 90% of cases exhibiting functional inactivation of the tumor suppressor genes TP53 and RB1 (32, 33). Other frequently mutated genes include SOX2, CREBBP, NOTCH1, EP300, TP73, SLIT2, PTEN, FGFR1, and MYC. In 1985, Carney et al. (34) identified significant molecular heterogeneity in human SCLC cell lines. They classified them into two major categories based on tumor cell biology and neuroendocrine (NE) phenotype: NE stem cell-like classic type and non-NE stem cell-like variant type. The former exhibits high neuroendocrine features (High NE), while the latter shows low neuroendocrine features (Low NE). The latter appears to be more malignant, more aggressive, and less radiosensitive. In 2019, Professor Rudin (35) first proposed a classification of SCLC into four subtypes based on the differential expression of four key transcription factors—ASCL1, NEUROD1, POU2F3, and YAP1: SCLC-A, SCLC-N, SCLC-P, and SCLC-Y. Among these, SCLC-A and SCLC-N belong to the neuroendocrine subtypes, while SCLC-P and SCLC-Y are non-neuroendocrine subtypes. A 2025 review from the University of Texas MD Anderson Cancer Center (36) comprehensively summarized the development of immunotherapy in SCLC. In 2021, Gay et al. (16) suggested that the previously proposed fourth molecular subtype, SCLC-Y, driven by YAP1, lacked confirmation. Instead, a study based on RNA sequencing and IHC provided evidence for an SCLC-I subtype, characterized by low expression of all four transcriptional regulators (ASCL1, NEUROD1, POU2F3, and YAP1). SCLC-I can also be termed the quadruple-negative subtype (SCLC-QN) and exhibits both an inflammatory gene signature and mesenchymal features (37). Researchers found that the SCLC-I subtype might be more sensitive to immunotherapy, potentially serving as a predictive factor. Concurrently, the tumor immune microenvironment (TIME) and liquid biopsies (e.g., ctDNA) are gradually being used to identify sensitive subpopulations, paving the way for precision medicine (38).

Since PD-L1 expression and TMB have not yet been established as mature predictive biomarkers for immunotherapy in SCLC patients, we further investigated the more economically feasible alternative of SF. As ferroptosis occurs within tumor tissues, this may have introduced some influence on our analysis (39). Elevated SF levels could be affected by local release from the tumor site, and SF levels decrease by approximately 50% after surgical resection of the tumor in patients. This suggests that the increase in SF may be associated with tumor mass (40). During the initial phase of SCLC treatment, SF levels drop significantly in most treatment-sensitive patients, paralleling the rapid reduction in tumor burden. A novel aspect of this study is the association of SCLC molecular subtypes with SF levels, further validating the predictive value of SF levels across different molecular subtypes by assessing patient response to immunotherapy. As a biomarker, serum ferritin offers several advantages compared to PD-L1 and TMB. First, sample collection and detection are straightforward, requiring only peripheral blood draws, thus avoiding the need for repeat tissue biopsies. Second, the cost of serum ferritin testing is relatively low, significantly less than that for PD-L1 and TMB, making it more accessible and acceptable for patients. Third, SF levels can be measured repeatedly to minimize the impact of laboratory errors on interpretation.

We fully agree that immunohistochemical staining for ferritin heavy chain (FTH1) and transferrin receptor (TfR1) on patient tumor samples would provide critical insights into whether serum ferritin levels truly reflect intratumoral iron metabolism and ferroptosis potential. In response, we have already initiated a pilot study using archived tumor tissue specimens from a subset of patients in our cohort (n=50, including both responders and non-responders, and representing a range of baseline SF and ΔSF values). These samples will be subjected to IHC staining for FTH1 and TfR1, and the expression levels will be correlated with serum ferritin parameters and clinical outcomes. Based on our clinical findings, we hypothesize that: Serum ferritin levels will correlate positively with tumoral FTH1 expression, suggesting that systemic iron overload is associated with increased iron storage within tumor cells, which may promote proliferation and resistance to ferroptosis.

An important consideration arising from our findings is the dual role of iron in cancer biology. Iron is a double-edged sword in the context of immunotherapy: while it is an essential cofactor for ferroptosis—a form of regulated cell death increasingly recognized as a key effector mechanism of immune checkpoint inhibitors which excess systemic iron can paradoxically promote tumor progression and immune evasion (41). Ferroptosis is driven by iron-dependent accumulation of lipid peroxides, and adequate intracellular iron levels are necessary for this tumor-suppressive mechanism to occur (42). However, epidemiological and preclinical studies have consistently shown that elevated body iron stores are associated with increased cancer risk and poorer outcomes (43).

This dichotomy may explain our observation that patients with extremely high baseline SF levels (>900 μg/L) exhibited particularly poor responses to immunotherapy. In these individuals, the tumor-promoting effects of iron overload which including enhanced cancer cell proliferation and support for immunosuppressive cell populations such as myeloid-derived suppressor cells (MDSCs) and M2-polarized macrophages—may outweigh any potential benefit from ferroptosis-mediated killing. Conversely, the favorable outcomes associated with high ΔSF (≥15%) during treatment may reflect a reduction in this systemic iron burden, thereby restoring a more favorable balance toward ferroptosis sensitivity and a less immunosuppressive microenvironment.

These findings suggest that the prognostic significance of SF is context-dependent, with both the absolute level and the direction of change during treatment providing important information. Future studies should investigate whether pharmacological modulation of iron homeostasis. For example, using iron chelators in patients with baseline iron overload could synergize with immunotherapy to improve outcomes in ES-SCLC. Additionally, translational studies examining the relationship between SF dynamics, intratumoral iron content, and the composition of the immune microenvironment would help elucidate the mechanistic underpinnings of our clinical observations.

A limitation of this study is that SCLC molecular subtyping was performed solely using immunohistochemistry, which has certain inherent limitations. Where conditions permit, we intend to utilize RNA sequencing or proteomic technologies to measure the expression levels of key transcription factors to further substantiate our conclusions. As this study only preliminarily confirmed the correlation between pre-treatment SF levels, the SF change rate combined with the inflammation model, and the prognosis of SCLC immunotherapy, further evidence-based validation is still lacking. Our next steps will involve conducting prospective clinical studies and simultaneously validating the consistency of SF level changes in different tumor tissues using experimental animal models. We will compare the findings related to SF with existing prognostic indicators for tumor immunotherapy to further delineate its strengths and weaknesses. In our future research plans, we intend to conduct translational studies, including experiments on SCLC cell lines representing different molecular subtypes (A, N, P, Y). These experiments will involve treatment with ferroptosis inducers (e.g., RSL3 or Erastin) and measurement of lipid peroxidation, cell viability, and iron storage levels to directly assess whether intracellular iron status correlates with sensitivity to ferroptosis and immunotherapy. Such investigations would help bridge the gap between our clinical observations and the underlying biology, potentially leading to more precise patient selection and novel therapeutic strategies.

In addition, among the 425 patients, only 12 (2.8%) received palliative radiotherapy for oligoprogression while on immunotherapy maintenance. Given the very small number, we believe the impact on overall survival analysis is minimal. However, we acknowledge that this could be a confounding factor, and note that future studies with larger cohorts should consider stratifying by radiotherapy receipt.

## 4 Conclusions

In summary, we have demonstrated that ΔSF combined with a multidimensional inflammation model (NLR and LDH) can serve as a biomarker for predicting the efficacy of immunotherapy in ES-SCLC, although the underlying mechanisms require further investigation. Based on our current findings and future prospects, the ΔSF combined with the multidimensional inflammation model holds the potential to become a widely recognized predictive indicator for treatment efficacy, analogous to the predictive value of PD-L1 in NSCLC immunotherapy. This provides a reference for better identifying patients with ES-SCLC who are likely to benefit from immunotherapy in clinical practice. According to our findings, the molecular subtype of SCLC shows a certain correlation with the efficacy of immunotherapy. In the future, could measuring SF levels enable the precise selection of SCLC patients across different subtypes for treatment, thereby avoiding overtreatment? If this concept can be realized and applied clinically, it would be a significant benefit for patients with ES-SCLC. Furthermore, research indicates that the TIME and liquid biopsies (e.g., CTCs, ctDNA) are increasingly being used to identify subpopulations sensitive to immunotherapy. This area also warrants further validation in future SCLC research, potentially paving the way for more personalized therapeutic strategies.

## Data Availability

The datasets presented in this study can be found in online repositories. The names of the repository/repositories and accession number(s) can be found in the article/**Supplementary Material**.
