# Supplementary material for: Serum ferritin change rate combined with a multidimensional inflammation model for predicting efficacy and survival in extensive-stage small-cell lung cancer patients undergoing immunotherapy: a single-center, retrospective cohort study
Source: Front Immunol. 2026 Mar 11;17:1755851. doi: 10.3389/fimmu.2026.1755851 (PMC13013539; doi:10.3389/fimmu.2026.1755851)
Supplement: Supplementary file 1 [file Table1.docx]

**Supplementary Material**

**Table S1**. Subgroup Analysis of Objective Response Rate (ORR) in ES-SCLC Patients Undergoing Immunotherapy

| Subgroup | Total N | Responders (CR+PR), n (%) | Non-responders (SD+PD), n (%) |
| --- | --- | --- | --- |
| All patients | 425 | 235 (55.3%) | 190 (44.7%) |
| Sex |  |  |  |
| Male | 302 | 164 (54.3%) | 138 (45.7%) |
| Female | 123 | 71 (57.7%) | 52 (42.3%) |
| Molecular Subtype |  |  |  |
| SCLC-A | 218 | 112 (51.4%) | 106 (48.6%) |
| SCLC-N | 95 | 49 (51.6%) | 46 (48.4%) |
| SCLC-P | 72 | 48 (66.7%) | 24 (33.3%) |
| SCLC-Y | 40 | 26 (65.0%) | 14 (35.0%) |
| Pre-treatment SF |  |  |  |
| < 260 μg/L | 224 | 137 (61.2%) | 87 (38.8%) |
| > 260 μg/L | 201 | 98 (48.7%) | 103 (51.3%) |
